# Discussion on Vivisection vs. Anti-Vivisection1Before the nineteenth annual meeting of the American Microscopical Society, Pittsburg, Pa., August 19, 1896.

**Published:** 1896-12

**Authors:** V. A. Moore

**Affiliations:** Ithaca. N. Y., Formerly bacteriologist to Department of Animal Industries, Washington, D. C.


### DISCUSSION ON VIVISECTION VS. ANTI-VIVISECTION.[1]

By PROF. V. A. MOORE, Ithaca, N. Y.,
Formerly bacteriologist to Department of Animal Industries, Washington, D. C.

I INTENDED to prepare a paper for this meeting on the importance of animal experimentation, but on account of stress of work was unable to do so. It seems necessary to bring a few facts before the society, coming as I have from the hot-bed of this contest. It is difficult to tell just who started this bill originally, but it was presented by Senator Macmillan, of Michigan. It was brought by certain people belonging to the anti-vivisectionist society. They planned a bill entitled, A bill for the further prevention of cruelty to animals in the District of Columbia. It is not necessary to read this, as it is quite an extensive document. While they had not claimed that they wished to prevent animal experiments, the bill if passed will prevent further investigation of the government in this line.

In the first bill presented to this committee, the law required that licenses should be issued by the district commissioners to all people engaged in vivisection, and the district commissioners should exercise judgment as to who were fitted to perform these experiments, putting into the hands of the district commissioners the power to grant these licenses, which must be obtained for every experiment. These permits must be obtained five days before the performance of the experiments. You who are familiar with animal experimentation know that a delay of five days is sufficient to spoil all good that may possibly come from such work. You see it practically stops all work. In addition to that, these commissioners were to appoint a committee of three from the Humane Society, so-called, who were to inspect laboratory work at any time they choose. You must open the doors and explain the investigation or experiment and stop all work and make an elaborate report to the district commissioners whenever requested to do so. This bill came up before the district commissioners and was opposed with as much argument as possible, but to no avail. The anti-vivisectionists procured a very oratorical lawyer and statements of experiments made in France thirty or forty years ago, and were successful. They did modify it somewhat, so that the committee should be appointed by the president and not by the district commissioners. While it seemed unreasonable to think this bill could

1. Before the nineteenth annual meeting of the American Microscopical Society, Pittsburg, Pa., August 19, 1896.

be reported, it was, and I have here the report on the same. It shows that the senators were fair in incorporating a good portion of the argument in opposition to it. It is on the calendar of the United States Senate and may pass that body immediately after convening of Congress in December. The people anxious for this bill have circulated petitions and have secured signatures of a great many men of prominence, bishops, clergymen and physicians. One of these has written Bishop Hurst a letter, saying that it was presented to him to prevent vivisection in schools, and the like, in the District of Columbia, and we have found from consulting other people that they considered this bill of that character and not of the sweeping nature it really is. It is one of the most outlandish, unreasonable combinations of proposed legislation that I have ever read, and its influence can be seen from the fact that these men and women who in the State of Massachusetts tried to get similar legislation through and then admitted that, if they can get this bill through Congress, they can use it in getting state legislation, so that really the time is coming when this country will be in the same deplorable condition that England has been for so many years. It is our opinion that vivisection in public schools should not exist. Laws in many states prohibit that. There is a law covering that in the District of Columbia. We have letters and a report from the commissioner there that vivisection is not practised in the schools and it is not practised in the district schools anywhere, so far as I can learn. We do not want it, do not believe it humane and do not wish to use the life of an animal unless it is for a definite purpose. These remarks seem necessary to show just the condition and the only way in which this bill can be stopped. I think from reports that a large number of senators and representatives who have committed themselves to it, show that the average man is interested in this great question. The admission of such laws has cost England millions of dollars. For example, two diseases have cost England in money $400,000,000. The experiments necessary to find out the causes of these diseases and to find out how these diseases could be prevented would cost comparatively little. Think of the number of animals that have died from these diseases, to the amount in value of $400,000,000, and in this country we had about the same thing going on until this method was devised. All the diseases which have caused innumerable millions of lives are now rare occurrences, such as small-pox, cholera, and like diseases. It seems to me the number of animals that have been sacrificed is very few. On the other hand, I believe the animals used in these experiments suffer inconsiderably. It is only a question of premature death; but the question, it seems to me, does not rest on that. We have vivisection or animal experimentation for three legitimate purposes. The first is the demonstration of facts already known.

I believe it is necessary to experiment in medical schools. I believe it is necessary, and think every physician in this audience will agree with me, that every medical student should perform experiments on animals. Nobody, in my opinion, has a right to perform surgical operations on the human body until he is familiar with these experiments on the animal, which is always done with anesthetics.

Then there is the necessity for experimentation for the determination of facts, in which the knowledge to be gained has not any immediate utilitarian value, but may be of untold benefit ultimately. Some people think it is wrong to sacrifice life for this purpose.

Experiments made years ago determined facts that were looked upon as ultra-scientific and that are now indispensable to our knowledge. We are in possession of them because those men experimented for fact's sake and nothing else. We make these experiments for the ultimate purpose of determining facts which will save life and alleviate suffering.

There has been circulated recently, emanating from Philadelphia, a paper having a great deal of influence, saying that there is not any such thing as rabies, and that as a specific disease it does not exist. I do not know how many are familiar with the Pasteur method, but I venture to give a brief explanation of this. It was found by Pasteur and many others that if rabbits or other animals are inoculated with rabies they will die with certain paralytic symptoms. Then, if we inoculate rabbits with healthy brains they remain well. Before this discovery by Pasteur, of people bitten by mad dogs, about 48 per cent. died. This has been reduced by Pasteur's treatment to less than 1 per cent. This disease exists in this country to an enormous extent. We have had in the city of Washington fifteen cases of rabid dogs during the last ten months. One lady who was bitten by her dog died of rabies. The period of inoculation in a rabbit is less than in a human being. This lady was bitten in the face very badly, on the arm, shoulder and neck, by a dog, and the dog was brought to us and I inoculated two rabbits, and on the fifteenth day the physician who had charge came to me saying, "she is showing signs of rabies." We examined the rabbits and they appeared perfectly well. She died that night and the rabbits two days later. Again, a lady brought a dog for examination, wrapped up in a blanket on her arm, saying it had indigestion. I inoculated rabbits and they developed rabies and died on the twelfth day.

I wish to make a motion that the president appoint a committee from this society to draw up resolutions protesting against this or any other bill prohibiting animal experimentation.

The following resolutions were reported to the society on Thursday, August 20, 1896, and unanimously adopted by a rising vote :

WHEREAS, The American Microscopical Society, assembled in Pittsburg, Pa., August 18, 1896, for its nineteenth annual meeting, recognises the fact that the development of biological and medical sciences has resulted largely from experiments upon living animals ; and

WHEREAS, Those engaged in experimenting upon living animals in scientific laboratories do so for the purpose of gaining the knowledge which may result in alleviating human suffering and improving the condition and value of domesticated animals ; and

WHEREAS, No evidence has been presented by those who advocate restrictive legislation showing that abuses, such as cruel and unnecessary experiments, are practised in the District of Columbia ; and

WHEREAS, Results of great scientific and practical importance have been obtained by experiments on the lower animals made in Government laboratories in the District of Columbia ; therefore be it

*Resolved*, That the American Microscopical Society earnestly protests against the passage of Senate Bill No. 1552, entitled, A Bill for the Further Prevention of Cruelty to Animals in the District of Columbia, or any modification of this bill, or of any other bill restricting animal experimentation for scientific purposes.

*Resolved*, That copies of these resolutions, attested by the signatures of the officers of this society, and of its committee appointed to draft these resolutions, be sent to the chairman of the committee on the District of Columbia in the United States Senate and House of Representatives.

(Signed) A. CLIFFORD MERCER, *President.*
WM. C. KRAUSS, *Secretary.*
MAGNUS PFLAUM, *Treasurer.*
V. A. MOORE, Ithaca, N. Y., *Chairman.*
D. S. KELLICOTT, Columbus, O.
SUSANNA PHELPS GAGE, Ithaca, N. Y.
EDITH J. CLAYPOLE, Wellesley, Mass.
HERMAN VON SCHRENK, St. Louis, Mo.

